# Neural-mesodermal progenitor interactions in pattern formation: an introduction to the collection

**DOI:** 10.12688/f1000research.5657.1

**Published:** 2014-11-14

**Authors:** Chaya Kalcheim, Kate G. Storey

**Affiliations:** 1 Department of Medical Neurobiology, Hebrew University of Jerusalem, Jerusalem, Israel; 2 College of Life Sciences, University of Dundee, Dundee, UK

## Abstract

Mesodermal and spinal cord progenitors originate from common founder cells from which they segregate during development. Moreover, neural and mesodermal tissues closely interact during embryogenesis to ensure timely patterning and differentiation of both head and trunk structures. For instance, the fate and morphogenesis of neural progenitors is dependent on signals produced by mesodermal cells and vice-versa. While some of the cellular and molecular signals that mediate these interactions have been described, much more remains to be uncovered. The scope of this collection will cover these interactions between neural (CNS or PNS) and mesodermal progenitors in patterning body plans and specific body systems in vertebrate embryos. This includes, but is not limited to, interactions influencing the formation of body axes, neural tube formation, neural crest migration, gut development, muscle patterning and myogenesis.

## Editorial

Clonal analysis indicated that trunk neural and mesodermal progenitors share a common lineage at an early developmental stage ( Tzouanacou *et al.*, 2009). These dual-fated neuromesodermal precursors then segregate and further differentiate into either spinal cord or somite precursors. With the aim of elucidating how these fates bifurcate, researchers recently set out to recapitulate their differentiation pathways *in vitro* departing from embryonic stem cells ( Gouti *et al.*, 2014). Notably, the above lineages born from common precursors exhibit close interactions during later development that play pivotal roles in patterning body plans and specific body systems. For example, graded mesodermal signals control the timing of neuronal differentiation in the spinal cord ( Wilson *et al.*, 2009); somitic signals control the proliferation, delamination, segmental migration and patterning of neural crest-derived peripheral ganglia and, reciprocally, neural crest cells pattern the organization of mesodermal derivatives (i.e; muscles) in both the head and trunk ( Kalcheim, 2011).

Thus, the cross-talk between these developing systems is highly dynamic and reiterative. Emerging data highlight additional interactions between mesoderm and neural progenitors in development of gut innervation, craniofacial patterning, etc. and much more is yet to be discovered.

This collection aims to be a platform for original reports, review articles and opinions that expand our understanding on tissue patterning during development including the generation of these tissues and the molecular nature of their interactions.
